# Suppression of the necroptotic cell death pathways improves survival in *Smn*^2*B*/−^ mice

**DOI:** 10.3389/fncel.2022.972029

**Published:** 2022-08-03

**Authors:** Lucia Chehade, Marc-Olivier Deguise, Yves De Repentigny, Rebecca Yaworski, Ariane Beauvais, Sabrina Gagnon, Niko Hensel, Rashmi Kothary

**Affiliations:** ^1^Regenerative Medicine Program, Ottawa Hospital Research Institute, Ottawa, ON, Canada; ^2^Department of Cellular and Molecular Medicine, University of Ottawa, Ottawa, ON, Canada; ^3^Center for Neuromuscular Disease, University of Ottawa, Ottawa, ON, Canada; ^4^Department of Pediatrics, Children's Hospital of Eastern Ontario, Ottawa, ON, Canada; ^5^Department of Medicine, University of Ottawa, Ottawa, ON, Canada; ^6^Department of Biochemistry, Microbiology, and Immunology, University of Ottawa, Ottawa, ON, Canada

**Keywords:** neuroinflammation, necroptosis, motor neuron disease (MND), multi-system disease, mouse genetic models

## Abstract

Spinal muscular atrophy (SMA) is a monogenic neuromuscular disease caused by low levels of the Survival Motor Neuron (SMN) protein. Motor neuron degeneration is the central hallmark of the disease. However, the SMN protein is ubiquitously expressed and depletion of the protein in peripheral tissues results in intrinsic disease manifestations, including muscle defects, independent of neurodegeneration. The approved SMN-restoring therapies have led to remarkable clinical improvements in SMA patients. Yet, the presence of a significant number of non-responders stresses the need for complementary therapeutic strategies targeting processes which do not rely solely on restoring SMN. Dysregulated cell death pathways are candidates for SMN-independent pathomechanisms in SMA. Receptor-interacting protein kinase 1 (RIPK1) and RIPK3 have been widely recognized as critical therapeutic targets of necroptosis, an important form of programmed cell death. In addition, Caspase-1 plays a fundamental role in inflammation and cell death. In this study, we evaluate the role of necroptosis, particularly RIPK3 and Caspase-1, in the *Smn*^2*B*/−^ mouse model of SMA. We have generated a triple mutant (TKO), the *Smn*^2*B*/−^; *Ripk3*^−/−^; *Casp1*^−/−^ mouse. TKO mice displayed a robust increase in survival and improved motor function compared to *Smn*^2*B*/−^ mice. While there was no protection against motor neuron loss or neuromuscular junction pathology, larger muscle fibers were observed in TKO mice compared to *Smn*^2*B*/−^ mice. Our study shows that necroptosis modulates survival, motor behavior and muscle fiber size independent of SMN levels and independent of neurodegeneration. Thus, small-molecule inhibitors of necroptosis as a combinatorial approach together with SMN-restoring drugs could be a future strategy for the treatment of SMA.

## Introduction

Spinal muscular atrophy (SMA) is a devastating neuromuscular disease characterized by low levels of the ubiquitously expressed Survival Motor Neuron (SMN) protein. The SMN protein is produced by the *Survival motor neuron 1* (*SMN1*) gene located on chromosome 5q13 (Lefebvre et al., 1995). The genetic basis of SMA involves a mutation or homozygous deletion of the *SMN1* gene and retention of at least one copy of the paralogous gene *SMN2* (Lefebvre et al., 1995). The latter undergoes alternative splicing resulting in insufficient SMN protein to compensate for *SMN1* loss (Lorson et al., 1999; Monani et al., 1999). In most forms of the disease, patients will exhibit variable severity of progressive muscle weakness and atrophy (Sumner, 2006).

SMN depletion leads to the profound loss of motor neurons of the spinal cord (Lefebvre et al., 1997) and peripheral organ pathology including the muscle (reviewed in Shababi et al., 2014). While the underlying mechanisms are not fully understood, there is growing evidence of cell death pathway activation (Simic et al., 2000, 2007; Shafey et al., 2005; Tsai et al., 2006, 2008; Dachs et al., 2011; Mutsaers et al., 2011; Hayhurst et al., 2012; Martínez-Hernández et al., 2013; Fayzullina and Martin, 2014; Simon et al., 2017; Carrasco et al., 2019; Courtney et al., 2019; Deguise et al., 2020a; Weissman et al., 2021) and inflammation in several tissues in SMA (Deguise and Kothary, 2017; Deguise et al., 2017; Wan et al., 2018; Salucci et al., 2021). As such, investigating cell death mechanisms could shed light on the specific pathogenic mechanisms underlying motor neuron death and muscle atrophy.

There is mounting evidence that apoptosis plays a role in motor neuron death in patients and mouse models of SMA. In spinal cords, a significant number of neurons appear to be dying from apoptosis accompanied by dysregulation of apoptosis regulators, specifically p53 (Simic et al., 2000, 2007; Soler-Botija et al., 2005; Tsai et al., 2006, 2008; Simon et al., 2017). While p53 activity has been traditionally linked to apoptosis, numerous studies have identified p53 involvement in other forms of cell death including necroptosis (Wang et al., 2016). In fact, a recent study shows that p53 induction mediates neuronal death *via* necroptosis (Peek et al., 2022). SMN depleted muscles also display dysregulation of cell death pathways and apoptosis (Shafey et al., 2005; Dachs et al., 2011; Mutsaers et al., 2011; Martínez-Hernández et al., 2013). Importantly, cell death and defects in skeletal muscle precedes overt spinal cord pathology such as denervation and motor neuron loss (Fayzullina and Martin, 2014; Sheng et al., 2018). Beyond the neuromuscular system, a potential role of cell death is supported by digital and cutaneous necrosis in SMA infants (Carrasco et al., 2019; Weissman et al., 2021). Likewise, in the *Smn*^2*B*/−^ mice, a mouse model of severe SMA (Bowerman et al., 2012), spleen, tail, and ear necrosis are observed (Deguise et al., 2020a). Altogether, this suggests a scenario in which cell death mechanisms contribute to overall SMA pathology.

Cell death is known to play a role in the regulation of inflammation but may also result from inflammation (Yang et al., 2015). For example, in the central nervous system (CNS), microglial cells sample the environment and recognize pattern recognition receptors and cell damage-associated molecular patterns, triggering an inflammatory response and subsequent cell death (Schroder and Tschopp, 2010; Boucher et al., 2018). There is neuroinflammation resulting from defective microglial and astroglial cells in SMA that is suggested to contribute to the activation of cell death pathways (reviewed in Abati et al., 2020). These observations are not exclusive to motor neurons and have been made outside the CNS. As such, inflammation pathways may contribute to cell death in neurons, muscle, and other affected organs in SMA.

Necroptosis is a regulated form of cell death mimicking features of both necrosis and apoptosis. Under pathological conditions, upregulation of death receptor family ligands can sensitize cells in the CNS to necroptosis (Ransohoff, 2016; Dhuriya and Sharma, 2018). Necroptosis is mediated by the activation of receptor-interacting protein kinases (RIPK1 and RIPK3) leading to the phosphorylation of mixed lineage kinase domain-like protein (MLKL) (Rodriguez et al., 2016). RIPK3 has been identified as a core component of this pathway since it is critical for the oligomerization of MLKL that culminates with robust inflammation and the release of cell damage-associated molecular patterns (Dhuriya and Sharma, 2018; Zhu et al., 2018). Depending on the cellular environment, inflammasome activation will occur which associates with Caspase-1, leading to the cleavage of IL-1β into the mature form and thus the secretion of pro-inflammatory cytokines (Dhuriya and Sharma, 2018).

In seeking to understand the pathways that could lead to neuron and muscle death in SMA, we turned to investigate necroptosis, a disease process in which cell death and inflammation have been linked. Given the strong evidence of programmed cell death and inflammation occurring in SMA, we generated a mouse model to study the pathway by knocking out *Ripk3* and *Casp1*. RIPK3 is a crucial mediator of necroptosis and homozygous deletion of *Ripk3* should target the necroptosis pathway (Zhang et al., 2011; Rodriguez et al., 2016). Caspase 1 knockout provides significant protection against IL-1β mediated inflammation (Schroder and Tschopp, 2010; Boucher et al., 2018). Therefore, we generated a triple mutant model, namely the *Smn*^2*B*/−^; *Ripk3*^−/−^; *Casp1*^−/−^ mice (henceforth referred to as TKO mice). While we did not observe protection against motor neuron loss nor neuromuscular junction (NMJ) pathology, there was a significant increase in survival and motor function improvement in TKO mice compared to *Smn*^2*B*/−^ mice. Interestingly, we also observed larger muscle fibers without any significant changes in neurogenic muscle atrophy markers. Our study shows that necroptosis plays a role in muscle fiber size indicating an intrinsic mechanistic relevant in skeletal muscle development. Understanding this mechanism is important given the availability of small-molecule inhibitors of necroptosis which could be combined with SMN-restoring drugs as a novel approach in the early treatment of SMA.

## Materials and methods

### Animals

*Smn*^+/−^ mice were crossed to *Smn*^2*B*/2*B*^ mice to obtain *Smn*^2*B*/+^ and *Smn*^2*B*/−^ animals maintained on the C57BL6/J background (Bowerman et al., 2012; Eshraghi et al., 2016). The *Smn*^2*B*/−^ mice are a model of SMA and the asymptomatic heterozygous *Smn*^2*B*/+^ mice are used as controls in these experiments. *Casp1*^−/−^; *Ripk3*^−/−^ knockout mice (Shutinoski et al., 2020) were provided by Dr. Sad's laboratory (University of Ottawa, Ottawa, Canada). The *Smn*^2*B*/−^; *Casp1*^−/−^; *Ripk3*^−/−^ triple mutant mice (TKO) were derived after multiple generations of crossing *Smn*^+/−^; *Casp1*^−/−^; *Ripk3*^−/−^ and *Smn*^2*B*/2*B*^; *Casp1*^−/−^; *Ripk3*^−/−^ mice on the C57BL6/J background. Establishment of the line was confirmed by genotyping the DNA extracted from mouse ear biopsies using multiplex PCR (see Supplementary Table 1 for primers used). Both male and female mice were included in the studies. Mice were bred and housed at the University of Ottawa Animal Facility and cared for under protocol OHRI-3343 according to the Canadian Council on Animal Care.

### Survival and weight analysis

Mice were tattooed by Animal Care Facility staff between P4 and P6 for longitudinal assessment of survival, body weight change and motor function tests. Mice were weighed every 2 days. For longitudinal assessment of survival, mice were weaned at P21 into cages containing DietGel 76A (ClearH_2_O), a purified dietary supplement. Additional animals were sacrificed at P9 or P19 for tissue collection and analysis.

### Motor function tests

Spinal reflexes were assessed using the righting reflex test. Motor balance and coordination was assessed using the pen test. Forelimb and hindlimb muscle strength were measured using an inverted mesh grip test. Testing was administered in the following order: (1) Righting reflex, (2) Pen test, and (3) Inverted mesh/wire grip test on the same day, every 2 days. A maximum of 30 s on the pen test and 60 s on the inverted mesh grip was considered the maximal threshold. Three measurements were taken in succession and the average was recorded for analysis using the lab's previously reported protocols (Deguise et al., 2020b).

### Tissue processing and staining

Tissues for quantitative biochemical analysis were harvested from pre-symptomatic mice at P9 and symptomatic mice at P19 and preserved as previously described (Deguise et al., 2020b). For paraffin embedding, the *tibialis anterior* (TA) muscles were fixed for 24 h while livers, hearts, and spinal cords were fixed for 48 h in 1:10 diluted buffered formalin (Thermo Fisher Scientific, Waltham, MA) at 4°C and then transferred to 70% ethanol at 4°C until processing. All samples were processed, sectioned and stained at the Department of Pathology and Laboratory Medicine (University of Ottawa) as previously described (Deguise et al., 2020b). For NMJ analysis, the *transverse abdominal* muscle (TVA) was dissected and isolated under a dissection microscope according to protocol detailed in (Murray et al., 2014). Briefly, the TVA was incubated with TRITC conjugated alpha-bungarotoxin for 30 min at room temperature. Muscles were subsequently permeabilized with 2% (v/v) Triton X-100 (Sigma), and blocked at room temperature with 4% BSA, 1% (v/v) Triton X-100 in PBS. Tissues were then incubated overnight at 4°C in blocking reagent containing primary antibodies for neurofilament and synaptic vesicle protein 2 (Supplementary Table 2). The following day, tissues were incubated in secondary antibodies (Supplementary Table 2) for 2 h at room temperature in the same antibody diluent. Slides were mounted with fluorescent mounting medium (DAKO mounting media S302380-2). Images were acquired using a Zeiss Axio Imager M1 microscope mounted with a camera.

### Image analyses and quantification

Quantification of spinal cord and muscle histology was performed in a blinded fashion. N number refers to individual mice and is described in each figure legend. Representative images of H&E-stained sections were acquired using the ZEISS Axio Scan Z1 slide scanner for brightfield at different magnifications. Muscle fiber size was quantified using ImageJ. About 100–200 muscle fibers in randomly selected areas of the muscle were counted to ensure proper coverage. The area of each fiber was measured to calculate a mean fiber size for each animal. Quantification of motor neuron number and size was performed on a total of 5 sections separated by 10 sections each per animal from the H&E-stained lumbar spinal cord. To ensure proper counts, motor neurons were identified by location in the ventral horn, presence of a nucleolus, and cells having cross-sectional area >350 μ*m*^2^. For quantification of choline acetyltransferase (ChAT) positive motor neurons, lumbar spinal cords were dissected, postfixed in 4% paraformaldehyde overnight at 4°C, then prepared for cryosectioning as previously described (Deguise et al., 2020b). The number of ChAT-positive motor neurons per ventral horn was recorded for four different sections per animal, each separated by at least 100 μ*m* to avoid counting the same motor neuron twice. Only ChAT-positive motor neurons (diameter ≥20 μm) were counted. NMJs were quantified as either normal or displaying neurofilament accumulation, and endplates were noted as either occupied or unoccupied.

### Western blotting

Total protein was collected by homogenization of flash frozen whole spinal cord, liver, heart, *tibialis anterior* (TA), and hindlimb muscles in RIPA lysis buffer (Cell Signaling) in the presence of PMSF (Cell Signaling) and proteinase and phosphatase inhibitors (Abcam). Protein concentrations were determined using Pierce BCA Protein Assay kit (ThermoFisher). Thirty *ug* protein per sample was separated by 10% sodium dodecyl sulfate polyacrylamide gel electrophoresis. Proteins were transferred to a PVDF membrane (Immobilon-P, Millipore) and blocked for 1 h at room temperature in 5% (w/v) skim milk in TBST. Membranes were then incubated in primary antibodies and secondary antibodies (see Supplementary Table 2). Proteins were visualized using Pierce ECL Western Blotting Substrate (Thermo Scientific) and SuperSignal West Pico PLUS Chemiluminescent Substrate (Thermo Scientific). Protein quantification was by densitometric analysis using ImageJ software (Version 1.51, Free Java software provided by National Institutes of Health, USA) by taking the mean gray value of bands for the target protein, normalized to loading control (α-tubulin or GAPDH) from the same blot.

### RNA isolation and RT-qPCR

Total RNA from the TA muscle was extracted using Qiagen RNeasy Mini kit, quantified and reverse transcribed using RT^2^ first strand kit (Qiagen) according to the manufacturer's protocol. qPCR was performed in technical triplicates for each sample using mouse primers obtained from Bio-Rad targeting Atrogin-1 (Fbxo32), MuRF1 (Trim63) and Gapdh. Each qPCR reaction contained 300 ng of cDNA, 2x SsoFast EvaGreen (Bio-Rad), RNase/DNase-free water, and respective primers in a final volume of 20 μl. Three negative controls containing water instead of cDNA were included in every qPCR plate. Results were quantified using 2^−Δ*ΔCt*^ method. Results were normalized to Gapdh.

### Statistical analysis

All data were expressed as mean ± standard error of the mean. Survival data was represented with Kaplan-Meier survival curves and statistically significant differences between groups were evaluated using the Mantel-Cox test. One-way analysis of variance (ANOVA) with Tukey *post-hoc* test was used to distinguish differences between more than two groups when multiple comparisons were necessary. Matched two-way ANOVA with Geisser–Greenhouse correction was used with complete data sets. Matched two-way ANOVA with mixed effects model was performed where values were missing (in case of death) with Sidak's or Tukey's method for multiple comparisons depending on the characteristics of the dataset. Statistical tests were performed using GraphPad Prism V.9.0 (GraphPad Software, San Diego, CA). Significance was set at *P* < 0.05.

### Data availability

All data will be made available upon contacting the corresponding author.

## Results

### Generation of a mouse model to study necroptotic cell death in *Smn*^2*B*/−^ mice

**Figure 1:**
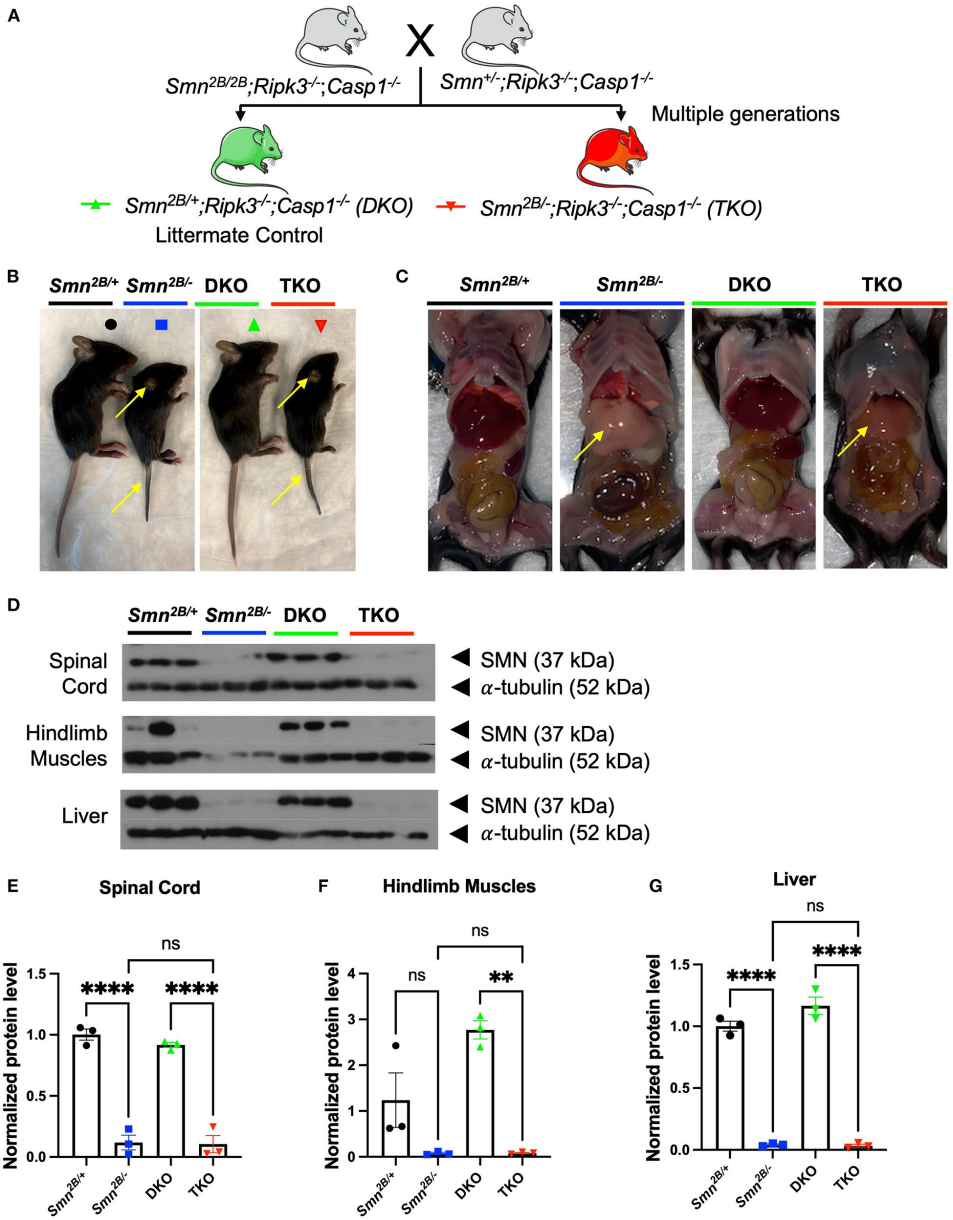
Generation of necroptotic cell death pathway knockout mice on the *Smn*^2*B*/−^ SMA mouse model background. **(A)** Schematic representation of the breeding scheme to generate the necroptotic pathway knockout on *Smn*^2*B*/+^ background (*Smn*^2*B*/+^; *Ripk3*^−/−^; *Casp1*^−/−^). These double knockout mice (DKO, Green) do not develop an SMA-like phenotype and serve as controls. *Smn*^2*B*/−^; *Ripk3*^−/−^; *Casp1*^−/−^ triple mutant mice (TKO, Red) harbor a necroptotic pathway knockout on the *Smn*^2*B*/−^ mouse background. Parental strains were generated by multiple generations of crossings. Mice were compared to the *Smn*^2*B*/+^ (control) and *Smn*^2*B*/−^ (mouse model of severe SMA). **(B,C)** Representative images of *Smn*^2*B*/+^ (Black), *Smn*^2*B*/−^ (Blue), DKO (Green) and TKO (Red) at postnatal day 19 (P19) and gross phenotypic changes as denoted by the yellow arrows pointing to tail, ear necrosis, and fatty liver. **(D)** SMN and α-tubulin immunoblots of *Smn*^2*B*/+^ control, *Smn*^2*B*/−^, DKO, and TKO mice spinal cord, muscle, and liver extracts. **(E–G)** Densitometric analysis of SMN western blots normalized to α-tubulin levels. Data represents means ± SEM with η = 4 per genotype. An ordinary one-way ANOVA with Tukey *post-hoc* test for multiple comparison was performed with *p* < 0.001 for **, *p* < 0.0001 for ****, and ns, non-significant.

To investigate necroptosis in an SMA mouse model, we generated a knockout of the necroptotic pathway in the *Smn*^2*B*/−^ mice. This was achieved *via* genetic crosses of mice to introduce the *Smn*^2*B*^ allele onto the *Ripk3*^−/−^; *Casp1*^−/−^ double knockout (Figure 1A). Two separate breeding schemes were initiated to obtain the *Smn*^+/−^; *Ripk3*^−/−^; *Casp1*^−/−^ and *Smn*^2*B*/2*B*^; *Ripk3*^−/−^; *Casp1*^−/−^ mice. These mice were paired and gave a progeny of triple mutant mice (TKO, *Smn*^2*B*/−^; *Ripk3*^−/−^; *Casp1*^−/−^) and littermate double knockout (DKO, *Smn*^2*B*/+^; *Ripk3*^−/−^; *Casp1*^−/−^) control mice with necroptotic pathway ablation (Figure 1A). The *Smn*^2*B*/+^ and *Smn*^2*B*/−^ mice were included as additional controls. Genetic background has a substantial effect on the phenotype and survival of severe mouse models of SMA (Eshraghi et al., 2016). Therefore, all mice used in this study were derived and maintained on a pure C57BL6/J background to exclude any potential effect related to a mixed genetic background. Reduced tail length and ear necrosis are two morphological canonical features present in the *Smn*^2*B*/−^ mice when compared to *Smn*^2*B*/+^ controls. These pathological features were preserved in the TKO mice compared to DKO mice (Figure 1B). Extra-neuronal pathology has been previously described in mouse models of SMA. In the *Smn*^2*B*/−^ mouse model, defects in fatty acid metabolism led to non-alcoholic fatty liver disease (NAFLD), which is a macroscopic pathological feature (Deguise et al., 2021). TKO livers were unchanged compared to the *Smn*^2*B*/−^ mice on gross morphology (Figure 1C) and histological evaluation confirmed that necroptotic pathway ablation does not impact fat accumulation in the liver (Supplementary Figures 1A–D). The *Smn*^2*B*/−^ mice express 15% of SMN protein in the spinal cord compared to wild type (Eshraghi et al., 2016), which we confirmed by western blot analysis (Figures 1D,E). Levels of SMN in TKO mice did not change from those observed in *Smn*^2*B*/−^ mice across all tissues assessed (Figures 1D–G). This means that any potential changes in phenotype are independent of SMN levels in TKO mice. Altogether, we conclude that genetic ablation of necroptotic cell death pathways does not alter SMN levels in TKO mice and that any phenotypic alterations may result from an SMN-independent mechanism.

### Genetic ablation of necroptotic cell death pathways prolongs survival in *Smn*^2*B*/−^ mice

**Figure 2:**
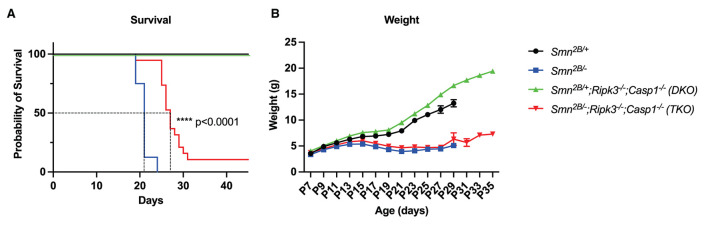
Impact of necroptotic pathway ablation on survival and weight gain in *Smn*^2*B*/−^ mice. **(A)** Kaplan–Meier curve comparing survival across genotypes up to 50 days displays increased survival in TKO mice compared to *Smn*^2*B*/−^ (*p* < 0.001). **(B)** Analysis of weight beginning at P7 to P35, shows a reduction in weight in TKO mice comparable to *Smn*^2*B*/−^. η = 11−19 per genotype. Data represents means ± SEM, statistical analysis **(A)** Mantel–Cox test and **(B)** An ordinary two-way ANOVA with Sidak's *post-hoc* test for multiple comparison. *P* < 0.0001 for ****.

TKO mice showed a mean increased survival of 6 days in comparison to the *Smn*^2*B*/−^ mice (median survival of 27 days compared to 21 days) (Figure 2A). In fact, by the time all the *Smn*^2*B*/−^ mice are dead, 95% of TKO mice remained alive, with a few animals surviving up to the very end of the observational period of 45 days (Figure 2A). The increased survival was independent of sex, since we could not observe a differential effect between male and female mice in the TKO mice (Supplementary Figure 2A). To determine whether increased survival correlates with improved weight gain, we assessed body weight every second day starting at P7. Similar to the *Smn*^2*B*/−^ mice, we noted a slowing down in weight gain in the TKO mice regardless of prolonged survival (Figure 2B).

**Figure 3:**
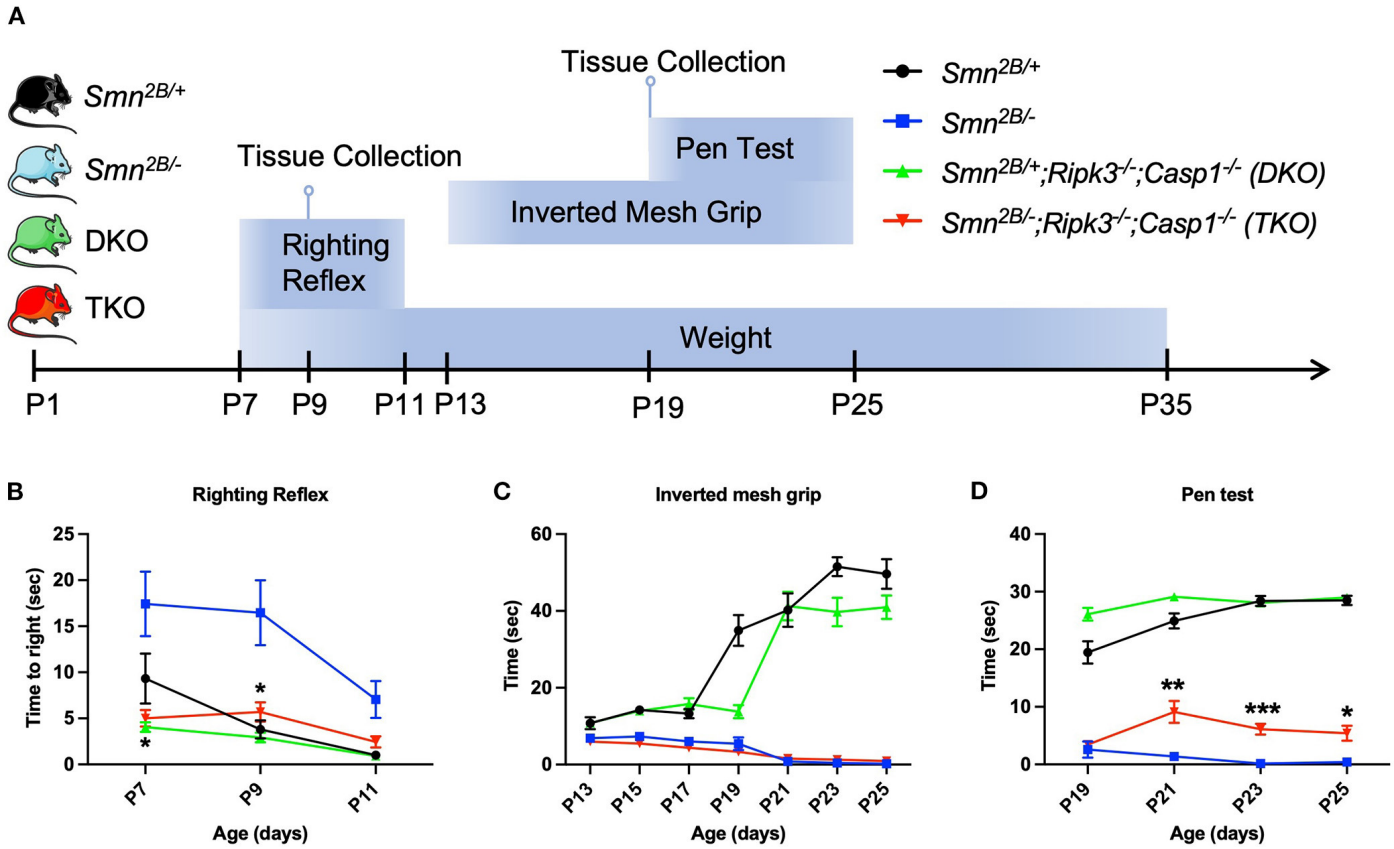
Impact of genetic ablation of necroptotic cell death pathways on motor function in *Smn*^2*B*/−^ mice. **(A)** Schematic representation of experimental design and evaluation of motor function. Motor function and weight were evaluated every second day in each group with **(B)** righting reflex from P7 to P11, **(C)** inverted mesh grip test from P13 to P25, and **(D)** pen test from P19 to P25. Minor motor improvement could be identified in the TKO mice on the righting reflex and the pen test while no improvement was seen on inverted mesh grip test. η = 11−19 per genotype. Data represents means ± SEM. An ordinary two-way ANOVA with Sidak's *post-hoc* test for multiple comparison. *P* < 0.05 for *, *P* < 0.001 for **, and *P* < 0.0002 for ***.

Qualitative observation revealed an increased mobility in TKO mice (data not shown). We therefore employed a battery of age-specific tests tailored to follow motor function in our mice (Figure 3A). Spinal reflexes, measured by the righting reflex, were slightly improved in TKO mice compared to *Smn*^2*B*/−^ mice at P7 and P9 (Figure 3B). In contrast, no changes were observed with the inverted mesh grip, which focuses on the distal muscle strength (Figure 3C). However, TKO mice did display significant improvements in motor function revealed by the pen test (Figure 3D). This test measures motor balance and coordination. The improved performance of TKO mice in this test is in line with the qualitative observations of a better gait. Altogether, these findings demonstrate that genetic ablation of necroptotic cell death pathways ameliorates survival and modestly improves some motor function in SMA mice.

### Genetic ablation of necroptotic cell death pathways does not protect against motor neuron loss or neuromuscular junction pathology in *Smn*^2*B*/−^ mice

**Figure 4:**
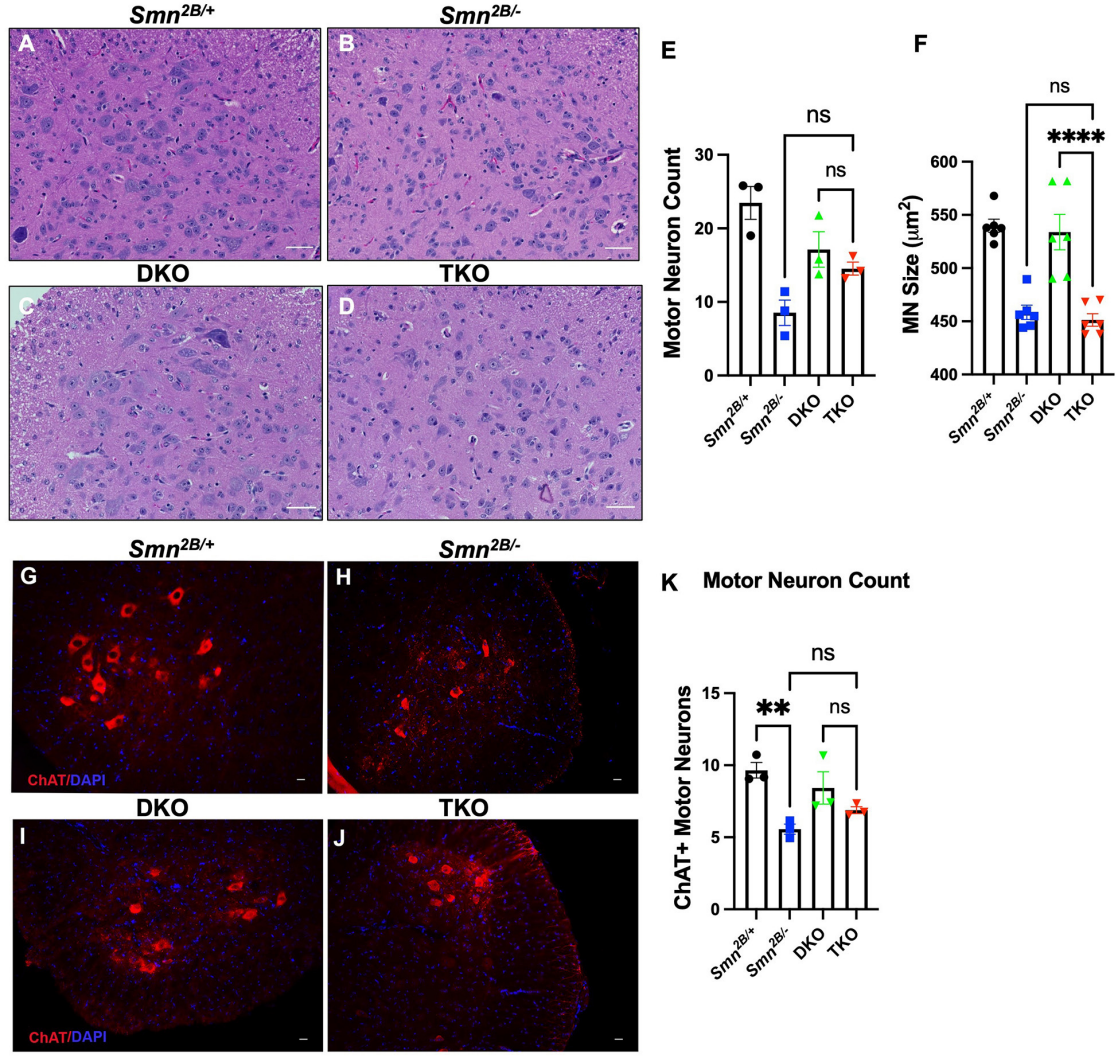
Genetic ablation of necroptotic cell death pathways does not rescue motor neuron degeneration in *Smn*^2*B*/−^ mice. **(A–D)** Representative H&E images (Scale bars = 50 μm) of sections of lumbar spinal cord from P19 mice and quantification of **(E,F)** motor neuron cell body number and size, identified by location in the ventral horn, presence of nucleolus, and cells bodies >350 μm^2^ in cross-sectional area. **(G–J)** Representative immunofluorescent images of lumbar spinal cord anterior horns stained for ChAT (red) and DAPI (blue) from P19 mice (Scale bars = 20 μm). **(K)** Quantification of ChAT positive motor neuron cell body number. The n value for each study is as follows; **(A–F)** η = 6 − 9 per genotype, **(G–K)** η = 3 per genotype. Note that a total of 5 sections separated by 10 sections each per animal from the H&E-stained lumbar spinal cord were analyzed for **(A–F)** and 4 sections separated by at least 100 μm per animal for **(G–K)**. Data represent mean ± SEM. An ordinary one-way ANOVA with Tukey *post-hoc* test for multiple comparison. ns, non-significant. *P* < 0.001 for **, and *P* < 0.0001 for ****.

**Figure 5:**
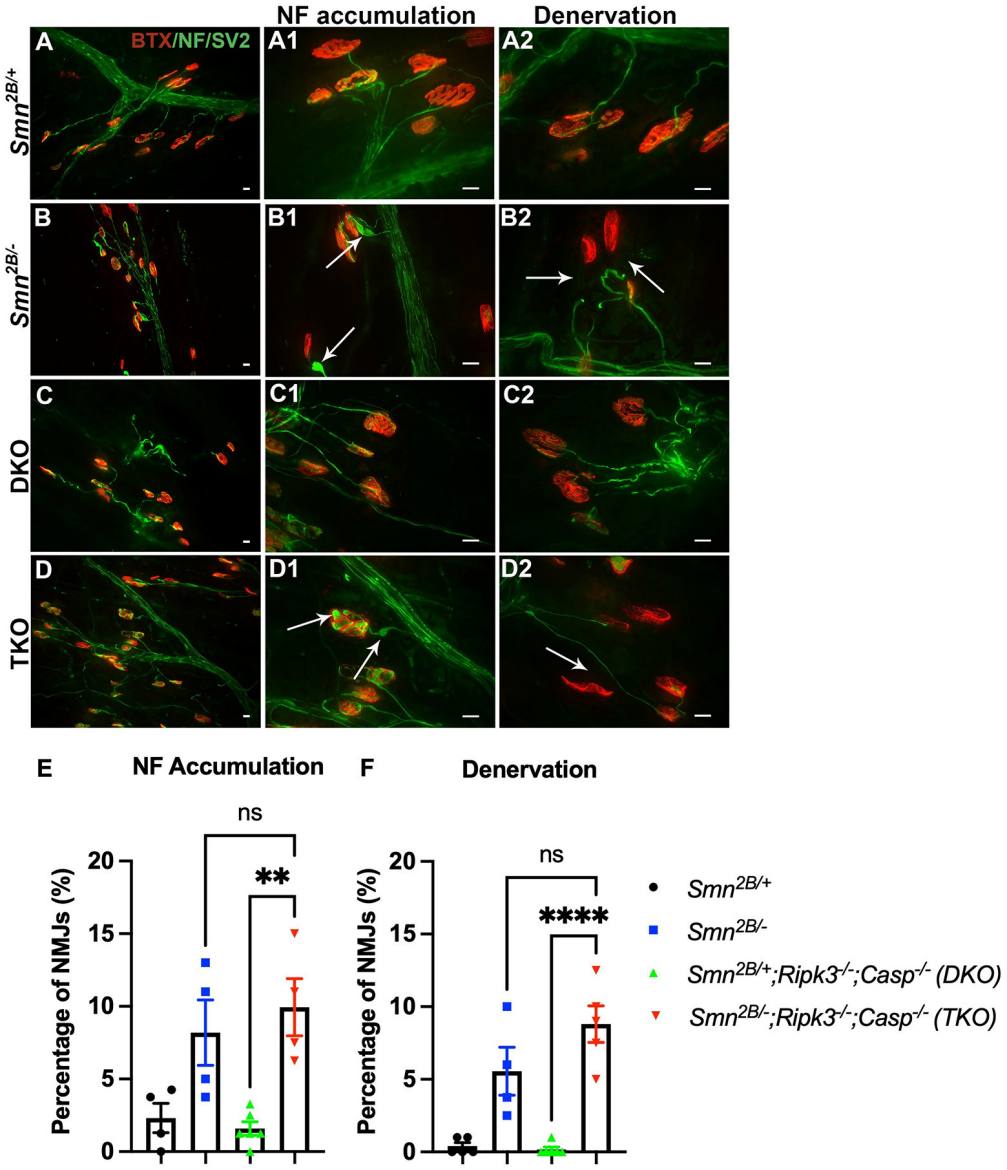
Genetic ablation of necroptotic cell death pathways does not improve NMJ pathology in *Smn*^2*B*/−^ mice. **(A–D)** Representative immunofluorescent images of the *transversus abdominis* (TVA) muscle stained with bungarotoxin (BTX; red) and for neurofilament (NF; green) and synaptic vesicle protein 2 (SV2; green). Quantification for NF accumulation **(E)** and denervation **(F)** in NMJs from P19 mice. White arrows show NF accumulation in **(A1–D1)** and end plate denervation in **(A2–D2)**. Scale bars = 20 μm. η = 4−6 per genotype. Data represent mean ± SEM. An ordinary one-way ANOVA with Tukey *post-hoc* test for multiple comparison. ns, non-significant. *P* < 0.001 for **, and *P* < 0.0001 for ****.

Next, we investigated whether attenuation of motor neuron loss and NMJ pathology, classical hallmarks of SMA, underly the improved motor function in TKO mice. H&E staining of the lumbar spinal cord did not show any changes in motor neuron number in TKO mice compared to *Smn*^2*B*/−^ animals (Figures 4A–E). This was further validated *via* choline acetyltransferase (ChAT) immunostaining, a specific marker of motor neurons (Figures 4G–K). No difference between DKO and TKO motor neuron count was seen in both ChAT and H&E-stained lumbar spinal cord sections. Perhaps this is due to the tendency for a decrease in DKO motor neuron count when compared to *Smn*^2*B*/+^ animals, and a tendency for an increase in motor neuron count between TKO and *Smn*^2*B*/−^ mice. Since we cannot explain this observation, we measured motor neuron size as an outcome for degeneration and we see no changes in motor neuron size in TKO mice compared to *Smn*^2*B*/−^ animals (Figure 4F). NMJ pathology has been shown to precede motor neuron death in *Smn*^2*B*/−^ mice (Buettner et al., 2021). Given that NMJ pathology is another readout for assessing the degeneration of the neuromuscular system in the *Smn*^2*B*/−^ mice, we quantified neurofilament accumulation and NMJ denervation (Figure 5). A comparison of *Smn*^2*B*/−^ with TKO animals revealed no difference in neurofilament accumulation or end plate denervation (Figures 5E,F). Altogether, we conclude that necroptosis pathway ablation does not influence the expected neuromuscular phenotype in SMA.

### Muscle fiber size is improved independently of muscle atrophy pathway in *Smn*^2*B*/−^ mice

**Figure 6:**
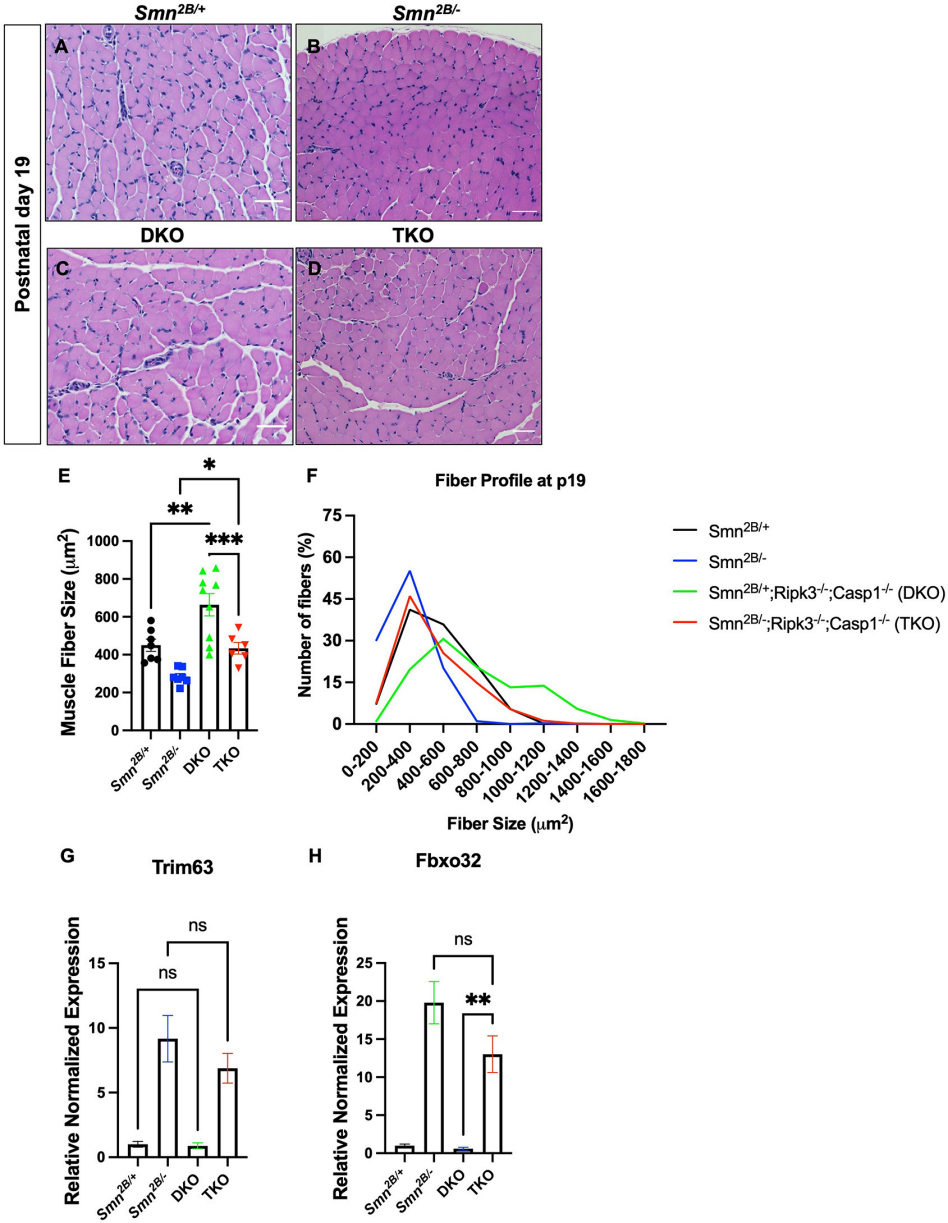
Genetic ablation of necroptotic cell death pathways increases muscle fiber size in both *Smn*^2*B*/+^ and *Smn*^2*B*/−^ mice. **(A–D)** Representative images of H&E-stained *tibialis anterior* (TA) muscle from P19 mice and **(E)** quantification of muscle fiber size area size. The fiber size profile distribution **(F)** is shifted to the right in the DKO and TKO muscle. Primers targeting Atrogin-1 (Fbxo32), MuRF1 (Trim63), and Gapdh were used to examine whether muscle fiber size was rescued due to reduction of muscle atrophy. **(G,H)** Levels of Atrogin-1 and MuRF1 mRNA are not significantly changed at symptomatic stage (P19) in the hindlimb of TKO mice compared to *Smn*^2*B*/−^. Scale bars = 50 μ*m*. The n value for each study is as follows; **(A–E)** η = 6 − 9 per genotype, **(G,H)** η = 4 per genotype. Data represent mean ± SEM. An ordinary one-way ANOVA with Tukey *post-hoc* test for multiple comparison. *P* < 0.05 for *, *P* < 0.001 for **, and *P* < 0.0002 for ***. ns, non-significant.

Given the lack of protection imparted by necroptosis at the motor neuron and NMJ level, we directly assessed muscle morphology in the TA muscle and whether muscle intrinsic mechanisms could be underlying the observed motor function improvements. Interestingly, TKO mice displayed larger muscle fibers compared to the *Smn*^2*B*/−^ mice at symptomatic stage (Figures 6A–E). Additional analysis of fiber size distribution revealed that there is an increased number of larger fibers in the TKO (Figure 6F). This effect is also observed in DKO mice when compared to *Smn*^2*B*/+^ controls, indicating an SMA/SMN independent effect. These findings suggest that genetic ablation of necroptosis pathways may modulate muscle intrinsic pathways involved in the regulation of muscle fiber size. Finally, muscle atrophy pathways have been previously shown to contribute to the pathophysiology of SMA (Deguise et al., 2016). Following denervation, the expression of the proteasomal E3 ligases, Atrogin-1 and MuRF1, is generally upregulated in *Smn*^2*B*/−^ mice compared to *Smn*^2*B*/+^ controls (Figures 6G,H). In TKO mice, mRNA levels of both Atrogin-1 and MuRF1 remained upregulated. Therefore, other mechanisms must be contributing to the amelioration of motor function in TKO mice.

## Discussion

Since there is mounting evidence that p53 plays a role in motor neuron death in different SMA mouse models (Simon et al., 2017; Courtney et al., 2019) and that p53 mediates neuronal cell death by necroptosis (Peek et al., 2022), it is important to investigate the contribution of necroptosis to motor neuron and muscle pathology in SMA. Initially, we attempted to quantify the expression of key mediators of necroptosis, RIPK1/RIPK3, MLKL, and Caspase-1, in the tissues of the *Smn*^2*B*/+^ and *Smn*^2*B*/−^ mice. Using, RT-qPCR analysis, we detected no change in the expression of RIPK1/RIPK3 and other mediators of necroptosis in the *Smn*^2*B*/−^ mice (data not shown). Further, we were unable to detect basal levels of these mediators nor their phosphorylated forms in tissue homogenates through western blotting. Our inability to detect any changes may be due to transient or temporal upregulation that we were unable to pick up given that our tissues were analyzed only at pre-symptomatic and symptomatic time points. Additionally, changes may be occurring at a cell-specific level that are difficult to pick up with analysis of whole tissue homogenates. Despite the lack of direct evidence in this model, it is relevant for the field to understand the impact of targeting necroptosis in a whole-body model.

In the present study, we generated a triple mutant (TKO) mouse of the necroptotic pathway in the *Smn*^2*B*/−^ mice, to gain a better understanding of inflammatory cell death by necroptosis in SMA. This model has an SMA phenotype comparable to the one in the *Smn*^2*B*/−^ mice. However, TKO mice had a notable increase in mean survival by 6 days, which is independent of any changes in SMN protein levels. This was accompanied by ameliorated motor function. However, necroptotic pathway ablation did not provide protection against motor neuron loss or NMJ pathology. Accordingly, markers of neurogenic atrophy remained elevated. Yet, we have identified muscle intrinsic changes following ablation of necroptosis, namely increased muscle fiber size in TKO mice.

Necroptosis has been shown to be involved in skeletal muscle pathology of other diseases (Bencze et al., 2018; Morgan et al., 2018; Kamiya et al., 2022). A first study in dystrophin-deficient muscles, identified RIPK3 as a key player in the degeneration of skeletal muscle (Morgan et al., 2018). Muscular dystrophy patients and dystrophin-deficient mice displayed an upregulation of RIPK3 which indicates necroptosis activation (Morgan et al., 2018). *Ripk3* ablation in the *mdx* mouse model improved motor function, which was attributed to a reduction in muscle degeneration, inflammation, and fibrosis (Morgan et al., 2018). In the context of inflammatory myopathies, suppression of necroptosis improved muscle weakness and cell death *via* reduction of pro-inflammatory cytokines and the NF-κβ pathway (Kamiya et al., 2022). As a result of necroptotic pathway ablation, apoptosis of muscle satellite cells was reduced (Kamiya et al., 2022). The beneficial effects of suppressing necroptosis thereby seem to be restricted to chronic muscle impairments since inhibition of necroptosis led to significant muscle regeneration defects in the context of acute toxin induced muscle injury (Zhou et al., 2020).

Here, we report an impact of necroptosis on SMA muscle which is independent of neuromuscular denervation. This indicates that necroptosis modulates muscle intrinsic defects in spinal muscular atrophy. This is supported by (i) findings of inflammation, (ii) dysregulation of muscle satellite cell homeostasis and (iii) increased apoptotic activity in SMA. While there appears to be no clear signs of muscle-intrinsic inflammatory processes, there is evidence of systemic inflammation. Reported inflammation across different tissues from SMA patients and mice (Deguise and Kothary, 2017; Deguise et al., 2017; Wan et al., 2018; Salucci et al., 2021) is suggested to aggravate atrophy of skeletal muscle and promotes cell damage. In muscle from SMA patients, smaller and disorganized myotubes are indicative of impaired growth and maturation (Martínez-Hernández et al., 2013). *In vitro* experiments in SMN-depleted muscle satellite cells, have identified satellite cell impairments, notably, accelerated differentiation and failure in forming myotubes (Le et al., 2011; Hayhurst et al., 2012). Moreover, apoptotic and cell death proteins are dysregulated in skeletal muscle from SMA patients and mouse models (Cifuentes-Diaz et al., 2001; Shafey et al., 2005; Dachs et al., 2011; Mutsaers et al., 2011; Martínez-Hernández et al., 2013; Armbruster et al., 2016).

Altogether, SMA patients and model mice exhibit systemic inflammation, impaired satellite cells, and skeletal muscle apoptosis, all of which can be modulated by necroptosis. In our study, muscle fiber size was improved in both DKO and TKO mice, implying an effect of necroptosis that is independent of SMA and SMN levels. This effect could be explained by reduced levels of pro-inflammatory cytokines and improved satellite cell function. Therefore, we suggest that necroptosis may have an indirect influence on muscle growth and maturation *via* reduction of pro-inflammatory cytokines and suppression of other forms of cell death happening in SMA muscle.

Preclinical models of severe SMA have a significantly reduced median lifespan of 10, 14, and 21 days in the *Taiwanese, SMN7* and the *Smn*^2*B*/−^ mouse models, respectively (Le et al., 2005; Gogliotti et al., 2010; Deguise et al., 2019). The remarkable increase in survival of TKO mice could relate to an amelioration of cell death in other tissues. Thus, effects of necroptosis on cell death pathways in other tissues besides muscle cannot be excluded. Abnormal apoptotic regulation is reported in spinal cord from SMA patients and mice (Simic et al., 2000, 2007; Soler-Botija et al., 2005; Tsai et al., 2006, 2008). Necrosis is also seen in cutaneous tissues in SMA infants (Araujo et al., 2009; Rudnik-Schöneborn et al., 2010; Carrasco et al., 2019; Weissman et al., 2021) and immune organs of *Smn*^2*B*/−^ mice (Deguise et al., 2017). Further, modulation of these cell death pathways alters survival, motor neuron loss, motor function, and muscle atrophy in mice (Tsai et al., 2006, 2008; Piras et al., 2017; Simon et al., 2017). Therefore, potential influences of necroptosis ablation in other tissues on these other reported cell death mechanisms cannot be ruled out. Besides, necroptosis has been described in chronic organ injury (Zhao et al., 2015). Peripheral organ defects are present in the *Smn*^2*B*/−^ mice, therefore the inhibition of necroptosis may protect against or attenuate damage to solid organs such as the kidneys, gut, lungs, and liver. Specifically, RIPK3 deficiency in an experimental model of NAFLD improved hepatic inflammation and hepatocyte injury without reducing the accumulation of fat in the liver (Afonso et al., 2021). As previously mentioned, the *Smn*^2*B*/−^ mice display a NAFLD phenotype. As such, a potential decrease in the expression of pro-inflammatory mediators and inflammatory cell infiltration in the livers of TKO mice resulting from a knockout of RIPK3 and Caspase 1, could contribute to the observed improved survival. Additional analysis is required to understand the underlying molecular mechanisms of necroptosis involved in protection of defective peripheral organs. In NAFLD, RIPK3 expression correlates with caspase-1 expression in the liver (Afonso et al., 2021). Therefore, RIPK3 knockout alone may be responsible for the seen effects in the TKO mice, however further work is required to tease out the contribution of each pathway in the TKO mice.

SMA is no longer considered the leading cause of infant mortality due to major advances in therapeutics which focus solely on restoring SMN levels (Kingsmore et al., 2020). Yet, SMN restoration does not fully rescue the phenotype, as seen in humans and animal mouse models (Gavrilina et al., 2008; Hua et al., 2011, 2015; Gogliotti et al., 2012; Reilly et al., 2022). Additionally, the mechanisms by which motor neuron loss and muscle atrophy occur in SMA remain unclear. Here, SMN levels in the TKO mice were the same to those measured in the *Smn*^2*B*/−^ mouse model. Yet, TKO mice had improved survival compared to the *Smn*^2*B*/−^ mice, a significant observation given the short lifespan of these mice. This means that our current findings are independent of SMN levels. While further research on the mechanism of necroptotic pathways in SMA is necessary, pharmacological inhibition could potentially serve as a treatment in combination with SMN-enhancing drugs.

## Conclusion

Cell death is an essential process in physiology, pathology, and development. Previous studies have exposed the contribution cell death by apoptosis, necrosis, and autophagy in SMA. However, no link to necroptosis has been previously made as a possible pathological mechanism contributing to CNS and peripheral pathology. Here, we report that targeting necroptosis pathways modulates survival, motor function and muscle fiber size independent of SMN levels and independent of the neurodegeneration. Commercially available small-molecule inhibitors of necroptosis such as Nec-1 could be combined with SMN-restoring drugs as a novel approach to ameliorate muscle function *via* modulation of muscle fiber size. Our results highlight the importance of further research to understand the mechanisms that could be modulated independently of SMN as a treatment for SMA.

## Data availability statement

The raw data supporting the conclusions of this article will be made available by the authors, without undue reservation.

## Ethics statement

The animal study was reviewed and approved by University of Ottawa Animal Facility.

## Author contributions

LC, M-OD, and RK designed the research. LC, M-OD, YD, and RY performed experiments. AB and SG provided support for experiments. LC, M-OD, and NH analyzed the data. LC, NH, and RK wrote the manuscript with input from all authors. All authors have read and agreed to the published version of the manuscript.

## Funding

RK was supported by Muscular Dystrophy Association (USA) (grant number 575466), Muscular Dystrophy Canada, and Canadian Institutes of Health Research (CIHR) (grant number PJT-156379). LC was supported by a CIHR Vanier Canada Graduate Scholarship. M-OD was supported by Fredrick Banting and Charles Best CIHR Doctoral Research Award.

## Conflict of interest

M-OD received honoraria and travel accommodations from Biogen for speaking engagements at the SMA Summit 2018 held in Montreal, Canada and SMA Academy 2019 held in Toronto, Canada. RK received honoraria and travel accommodations from Roche as an invited speaker at their global and national board meetings in 2019. RK and the Ottawa Hospital Research Institute have a licensing agreement with Biogen for the *Smn*^2*B*/−^ mouse model. This COI is outside the scope of this study. The remaining authors declare that the research was conducted in the absence of any commercial or financial relationships that could be construed as a potential conflict of interest.

## Publisher's note

All claims expressed in this article are solely those of the authors and do not necessarily represent those of their affiliated organizations, or those of the publisher, the editors and the reviewers. Any product that may be evaluated in this article, or claim that may be made by its manufacturer, is not guaranteed or endorsed by the publisher.
