# Supplementary material for: Suppression of the necroptotic cell death pathways improves survival in Smn2B/− mice
Source: Front Cell Neurosci. 2022 Aug 3;16:972029. doi: 10.3389/fncel.2022.972029 (PMC9381707; doi:10.3389/fncel.2022.972029)
Supplement: Supplementary file 1 [file Table_1.DOCX]

**Supplementary Table 1. List of primers used for genotyping and RT-qPCR studies.**

| **Primer name** | **Sequence** |
| --- | --- |
| Cas1 (Wt fwd) | GAGACATATAAGGGAGAAGGG |
| Cas 1 (Wt rev) | ATGGCACACCACAGATATCGG |
| Cas 1 (KO fwd) | TGCTAAAGCGCATGCTCCAGACTG |
| Rip3k (Wt fwd) | GGAGCCATTCTCCATGAATC |
| Rip3k (Wt rev) | AATCGTTCCTGGATGGTGAG |
| Rip3k (KO fwd) | GATCCTGATCCTGACCCTGA |
| Rip3k (KO rev) | ATCGACAAGACCGGCTTCCATCCGA |
