# Supplementary material for: Suppression of the necroptotic cell death pathways improves survival in Smn2B/− mice
Source: Front Cell Neurosci. 2022 Aug 3;16:972029. doi: 10.3389/fncel.2022.972029 (PMC9381707; doi:10.3389/fncel.2022.972029)
Supplement: Supplementary file 2 [file Table_2.DOCX]

**Supplementary Table 2. List of antibodies used for immunofluorescence studies.**

| **Method** | **Antibody** | **Species** | **Dilution** | **Company (Catalog #)** |
| --- | --- | --- | --- | --- |
| IHC | ChAT | goat | 1:100 | Millipore (AB144P) |
| IHC | anti-goat Alexa Fluor 555 | Donkey | 1:200 | Invitrogen (A21432) |
|  | TRITC conjugated bungarotoxin | N/A | 1:1 000 | Invitrogen (T1175) |
| IHC | Neurofilament (NF-M) | Mouse | 1:100 | (Developmental Studies Hybridoma Bank, P12839) |
| IHC | Synaptic vesicle glycoprotein 2A (SV2A) | Mouse | 1:250 | (Developmental Studies Hybridoma Bank, Q7L0J3) |
| IHC | Anti-mouse Alexa Fluor 488 | Goat | 1:250 | Invitrogen (A11001) |
| Western Blot | SMN | Mouse | 1:2 000 | BD Transduction (610647) |
| Western Blot | Alpha-tubulin | Rabbit | 1:10 000 | Abcam (ab4074) |
| Western Blot | Anti-mouse IgG HRP conjugate | Goat | 1:3 000 | Bio-Rad (1706516) |
| Western Blot | Anti-rabbit IgG HRP conjugate | Goat | 1:3 000 | Bio-Rad (1706515) |
