# Supplementary figures and images for: Suppression of the necroptotic cell death pathways improves survival in Smn2B/− mice
Source: Front Cell Neurosci. 2022 Aug 3;16:972029. doi: 10.3389/fncel.2022.972029 (PMC9381707; doi:10.3389/fncel.2022.972029)

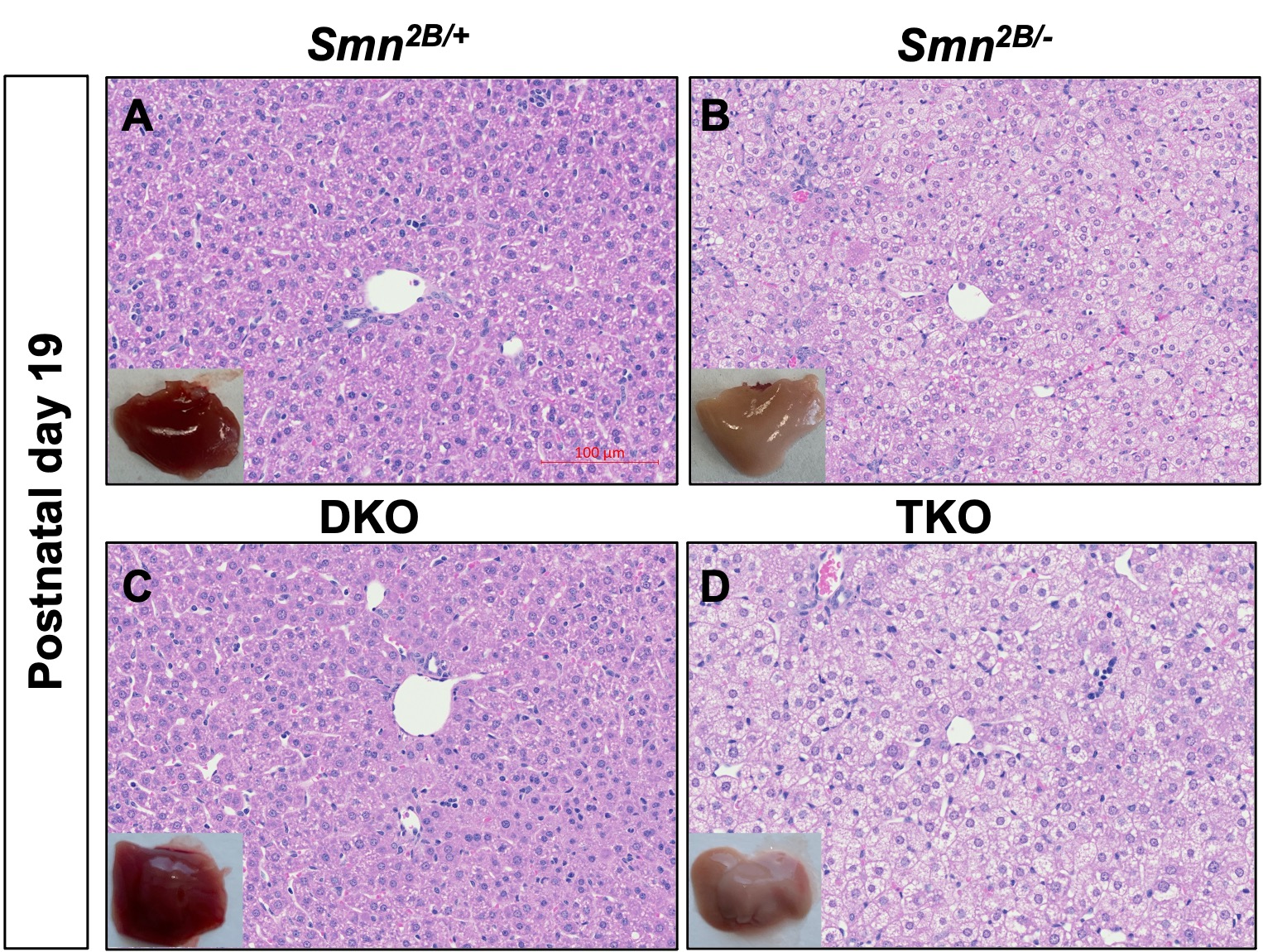

Supplement: Supplementary file 3 [file Image_1.JPEG]

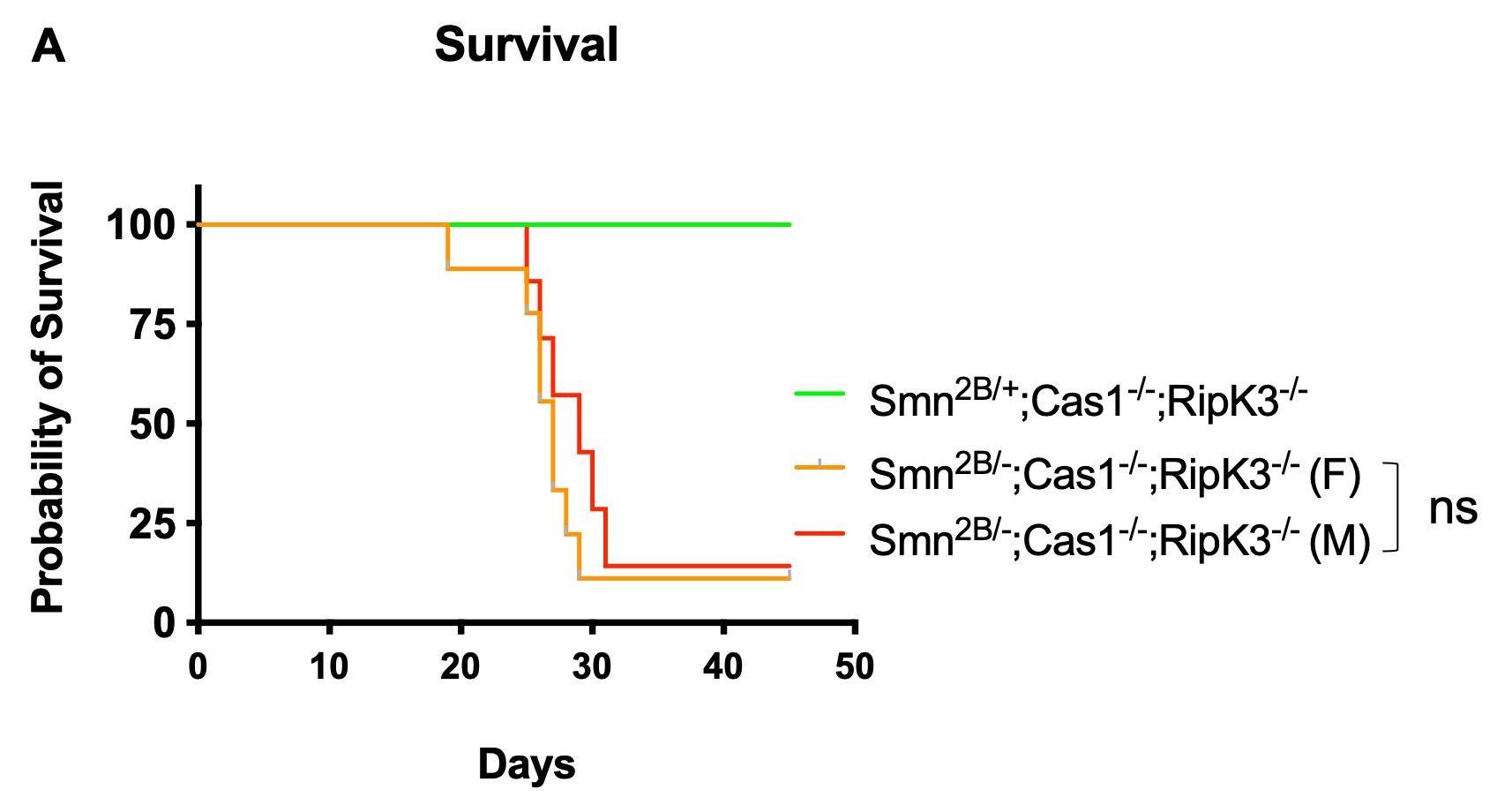

Supplement: Supplementary file 4 [file Image_2.TIFF]
